# A High-Density EEG Study Investigating VR Film Editing and Cognitive Event Segmentation Theory

**DOI:** 10.3390/s21217176

**Published:** 2021-10-28

**Authors:** Feng Tian, Hui Wang, Wanqiu Cheng, Wenrui Zhang, Yingjie Li

**Affiliations:** 1 Shanghai Film Academy, Shanghai University, Shanghai 200072, China; ouman@shu.edu.cn (F.T.); wanghui_@shu.edu.cn (H.W.); wanqiucheng@shu.edu.cn (W.C.); zhangrr233@shu.edu.cn (W.Z.); 2 Shanghai Film Special Effects Engineering Technology Research Center, Shanghai University, Shanghai 200072, China; 3 School of Life Sciences, Shanghai University, Shanghai 200444, China; 4 College of International Education, Shanghai University, Shanghai 200444, China; 5 Institute of Biomedical Engineering, Shanghai University, Shanghai 200444, China

**Keywords:** EEG, VR film, cognitive event segmentation theory, visual evoked potential

## Abstract

This paper introduces a cognitive psychological experiment that was conducted to analyze how traditional film editing methods and the application of cognitive event segmentation theory perform in virtual reality (VR). Thirty volunteers were recruited and asked to watch a series of short VR videos designed in three dimensions: time, action (characters), and space. Electroencephalograms (EEG) were recorded simultaneously during their participation. Subjective results show that any of the editing methods used would lead to an increased load and reduced immersion. Furthermore, the cognition of event segmentation theory also plays an instructive role in VR editing, with differences mainly focusing on frontal, parietal, and central regions. On this basis, visual evoked potential (VEP) analysis was performed, and the standardized low-resolution brain electromagnetic tomography algorithm (sLORETA) traceability method was used to analyze the data. The results of the VEP analysis suggest that shearing usually elicits a late event-related potential component, while the sources of VEP are mainly the frontal and parietal lobes. The insights derived from this work can be used as guidance for VR content creation, allowing VR image editing to reveal greater richness and unique beauty.

## 1. Introduction

With the rapid development of virtual reality technology, the integration of VR technology and film has gradually become an important breakthrough in traditional screen cinema [1], and VR films have shone at major film festivals, such as Venice, Sundance, and Golden Shaker. Immersive and interactive VR films might present people with the most extreme visual impact and sensory experience to date, enabling viewers to actively watch multi-threaded films while breaking numerous traditional rules of film shooting and editing.

In traditional cinema, filmmakers have developed a series of film editing rules for better transitions between scenes, collectively known as “continuity editing” [2,3]. According to the theory of cognitive event segmentation, editing connects clips shot at different times and places. Although the visual content might change dramatically according to different editing methods, viewers can effortlessly perceive the discontinuous flow of information as a series of coherent events [4], e.g., the 180-degree rule [5], which may smooth out the changes between scenes, and whose violation can cause confusion and discontent among the audience. However, the emergence and development of VR films have subverted and reconstructed traditional film narrative modes. The direction of the camera is controlled by the audience, the editing techniques, such as camera orientation and zoom, are no longer applicable in traditional films, and attention guidance becomes the editing method of VR films [6].

The focus of recent additional research has turned to the neural mechanisms behind films. Hasson et al. [7] also proposed the concept of Neurocinematography in 2008, where the impact of films on viewers can be measured by brain activity. Linking cinematography to cognitive neuroscience is a great impetus to find the connection between neuroscience and art. According to the current research, electrocardiogram (ECG) [1,8], functional magnetic resonance imaging (fMRI) [9,10], blood pressure (BP), and electroencephalogram (EEG) [11] can be used as assessment indicators. The electroencephalogram (EEG) is one of the oldest technologies to measure neuronal activity of the human brain [12]. The main neuroimaging techniques that can be used to study brain networks or network neuroscience include EEG, MEG, and MRI. The most prominent feature of EEG is the ultra-high temporal resolution compared to other imaging modalities (mainly compared to MRI techniques). EEG, as a non-invasive technical feature, could record cerebral evoked potentials from the skull surface to reflect neurophysiological changes in the brain during cognition, providing a reliable basis for studying information processing, such as attention, perception, emotion, movement, decision making, and judgment [13]. However, one of the fatal problems is that EEG has a severe volume conduction effect. To solve this problem, Pascual-Marqui used the standardized low-resolution brain electromagnetic tomography algorithm (sLORETA) traceability method to obtain the current distribution and intensity deeper in the cortex [14]. Lorenzo-López et al. used sLORETA to study EEG signals under a visual search task and found that neural activity in the anterior cingulate gyrus, limbic system, and occipitotemporal regions was lower in the group of older adults than the corresponding activity in the group of young adults [15].For conventional 2D films, it has been demonstrated that film clipping can cause certain physiological responses, such as lowered heart rate [16] and lowered blood pulse [13]. In addition, when Anderson [17] explored cortical activation patterns during viewing montage videos with the help of fMRI techniques, finding that viewing videos with continuous clips would activate temporal, parietal, occipital, and frontal regions of the brain, especially the right hemisphere regions. Experimental results by Heimann [10] showed that, relative to still shots, transporting the mirror could activate the brain’s sensory-motor areas and stimulate motor imagery even when filming still objects. SangHee et al. [18] compared sports, news, and advertising images in both 2D and VR environments, realizing that stronger beta wave vibrations are presented when viewing VR stereoscopic effects and fast-paced kinetic videos. Matranfernandez et al. [19] compared the EEG signals of subjects viewing 930 clips from five Hollywood movies and found that ERPs would produce larger amplitudes in longer scenes and differ in amplitude between and within movies, which is presumably related to editing techniques.

Less research has been done on VR movies, especially for VR film shot grouping, mainly on specific cinematography [20], shot guidance, perspective [21], depth of field changes [22], and compositing and rendering techniques [23], etc. Tricart Celine [24] provides a practical guide about using virtual reality in filmmaking in *Virtual Reality Filmmaking: Techniques & Best Practices for VR Filmmakers*, including narrative, documentary, and live event production, which covers the way to make a film in VR from beginning to end. Since traditional filmmaking techniques for directing audience attention are not directly applicable in VR films, practices such as panning or changing camera movements are no longer defined by the filmmaker, but by the audience. In this regard, some guidance methods for VR films have been proposed [25]. Syrett et al. [26] suggested that although there are some elements may distract the viewer in VR films environment, participants can generally follow the plot and characters. However, directing the viewer’s attention is still a challenge. Sylvia et al. [27] categorized these attention-guiding methods and provided a taxonomy based on the different characteristics. Katrin et al. [28] compared continuity edits and cuts-across the line events under VR and 2D conditions and found that jump cuts and nonlinear clips would usually cause ERP components in the early stage.

The purpose of this paper is to study factors affecting immersion and load by designing image stimulation experiments with different editing techniques in VR scenes, simultaneously collecting scalp EEG signals and analyzing their characteristics. Based on the clipping design in three dimensions, namely space, time, and action, this paper will analyze and compare the psychological and physiological characteristics according to different VR image stimuli.

In brief, the main contributions of this paper are as follows:

- Electroencephalograms (EEG) and visual evoked potential (VEP) analysis were performed, and the standardized low resolution brain electromagnetic tomography algorithm (sLORETA) traceability method was used to analyze the data.
- It was found that different editing techniques lead to different physiological and psychological indicators of viewers, while the physiological and psychological perception results tend to be the same.
- It is proven that the cognitive event segmentation theory also plays an instructive role in virtual reality editing. Even though VR movies are different from traditional movies in terms of presentation and viewing style to a certain extent, viewers’ perception of events in edited VR movies is similar to that of traditional photography. The experimental results will provide an experimental reference for VR movie research and necessary theoretical support for VR movie editing. Therefore, it has good academic and application value.

The rest of the paper is organized as follows. Section 2 describes the specific experimental procedure, data acquisition methods, and details the methods for pre-processing EEG data and classification. Section 3 presents detailed experimental results. A discussion is presented in Section 4. Finally, conclusions are made in Section 5.

## 2. Materials and Methods

To minimize experimental error, a pre-experiment was conducted before the formal experiment. The formal experiment consisted of a subjective questionnaire (NASA task load index (NASA-TLX) [29], an immersion questionnaire (IPQ) [30]) and a set of visually induced EEG-based experiments. The variables studied are hypothesized to affect the user’s perception of presence, spatial perception, and comfort of the experimental content, and to various degrees, but none of them will affect viewing.

### 2.1. The Participants

Briefly, thirty participants with an average age of 23.63 ± 1.33 years were recruited for this experiment, of whom 16 were male and 14 were female. There was no literature mentioning that the gender of normal adults would affect the content of this study. Thus, the effect of gender would be neglected in this experiment. The subjects were all Shanghai University students, all right-handed, with normal or corrected visual acuity and no history of psychiatric disorders. Before the formal experiment, participants were given 15 min to familiarize themselves with the experimental environment and operation, after which they were instructed to watch a video clip. All participants signed an informed consent form before the experiment and were given appropriate remuneration at the end of the experiment.

### 2.2. Experimental Materials and Hardware Equipment

**Figure 1:**
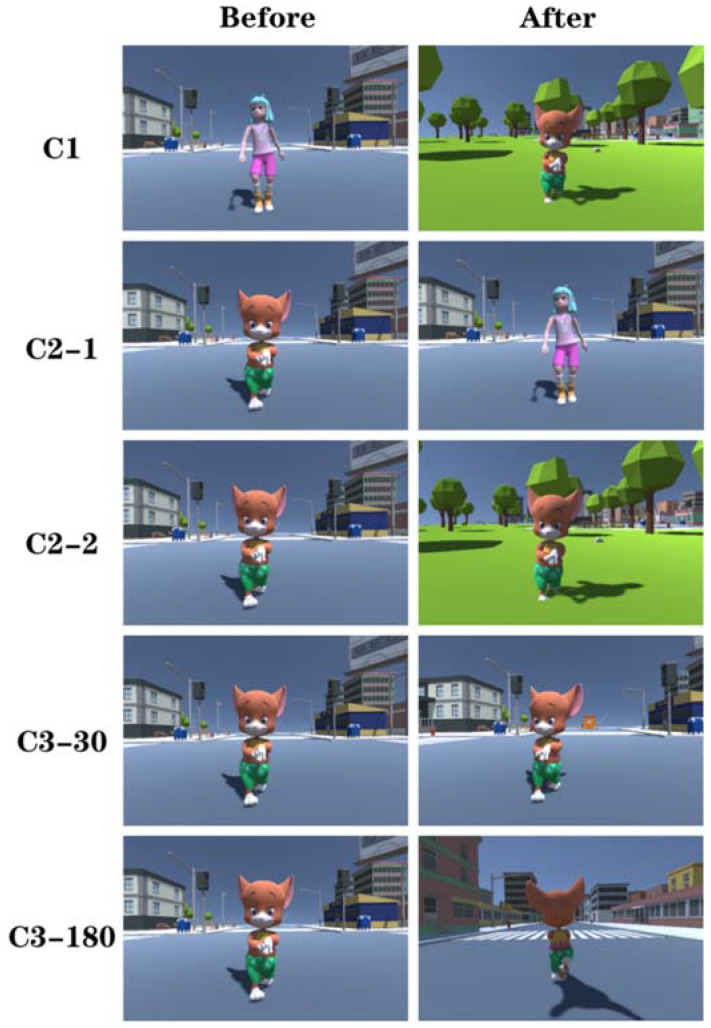
Shown are clips of the experimental material. The left (front) and right (back) are before and after the editing of each of the five editing types.

According to the event segmentation theory, a continuous domain in three dimensions of time, action (character), and space is defined, corresponding to C1, C2, and C3 separately. C1 indicates a discontinuity in space, time, and action, C2 illustrates a discontinuity in time or action (character), which is subdivided into C2-1 (continuous in time, discontinuity in action) and C2-2 (continuous in action, discontinuity in time). C3, which essentially refers to the change of viewpoint in the same scene is subdivided into 30° and 180° according to the angle of spatial change, denoted as C3-30 and C3-180 respectively (as in Figure 1). Note that C0 stands for the continuous video without clipping. Only 1 FOI under all conditions was found and it remains constant. Thus, there were 6 different conditions. Each condition corresponded to 25 scenes, resulting in a total of 150 stimuli. All experimental materials were produced using Unity 2018.4, post-processed with Adobe After Effects CC 2018. The video format was encoded with H.264, the resolution was 4096 ∗ 2048, and the frame speed was 30 frames per second.

The virtual reality environment was implemented in Unity 2018.4, running on a PC with a 3.4 GHz Intel Xeon E5-1230 V5 processor, 32 GB RAM, and an NVIDIA GTX 1070D. The PC monitor used was an AOC 24-inch LCD and the HMD headset monitor an HTC VIVE.

### 2.3. Experimental Procedure

The experiment was conducted in a closed, soundproof environment where the participant sat comfortably in front of a monitor, keeping his head dry and clean, and then wore a 64-channel EEG cap with channels distributed according to the International 10–20 System brain electrodes. Conductive paste was applied to the corresponding locations of the electrodes to reduce impedance, and attention was always paid to the signal transmission of each acquisition channel on the EEG cap. HTC VIVE HMD was needed to watch VR images.

**Figure 2:**
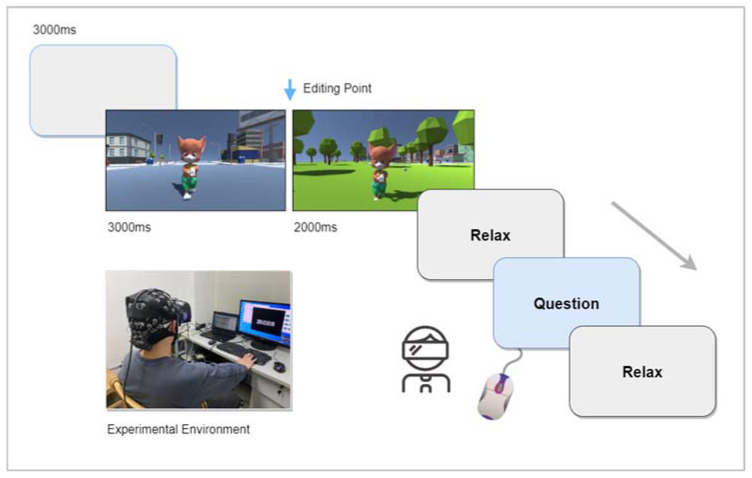
Experimental procedure.

To reduce noise interference, subjects were informed in advance that they should not talk nor clench their teeth and to minimize blinking during the viewing of the video. Before starting the experiment, a 3-min open-eye resting state signal was collected to familiarize the subjects with the experimental environment through a pre-experiment, and then the subjects were instructed to watch the corresponding images. The experimental procedure is shown in Figure 2. Before each new scene was viewed, participants were given 3 s to familiarize themselves with the scene to reduce the difference in the familiarity of each participant with the environment, followed by a stimulus image of 5 s in length. The subjective scale shall be filled out after each trial, and the order of presentation shall be randomly rotated between trials. The duration of viewing, scoring, and resting time was controlled by the subjects. The entire experiment took about 80 min.

### 2.4. Data Recording and Processing

The characteristic channels selected for statistical analysis of EEG were frontal region (Fz, F3, F4, FCz, FC3, FC4), parietal region (Pz, P3, P4), central region (Cz, C3, C4, CP3, CP4), temporal region (TP7, TP8, T7, T8, P7, P8), and occipital region (POz, PO3, PO4, Oz, PO7, PO8) [31]. The subjects’ EEG data were acquired via Neuroacle EEG Recorder V2.0.1, a toolbox of the software Matlab 2016 (Math Works, USA), with a sampling frequency of 1000 Hz and electrode impedances all less than 5 kQ. Data were preprocessed through EEGLab. Five useless channels were removed (AFz was used as the ground electrode, and two vertical and two horizontal EEGs were recorded; the electrode at T6 was damaged), filtered with a bandpass of 0.1–90 Hz, and then filtered with 50 Hz and 100 Hz trap filters to remove industrial frequency interference [32]. After the interpolation of bad leads and rejection of bad segments, oculoelectric artifacts were removed using the independent component analysis (ICA) method [33]. Depending on the task, the data were extracted by segmentation, selecting data from the scene before the editing point as the baseline of each stimulus segment, and excluding segmented data with obvious artifacts (wave amplitude > ±100 μV) [34]. Baseline drift was re-referenced [11] and eliminated. Chella et al. [35] showed that the error of this zero-reference method was significantly smaller than that of whole-brain averaging, bilateral mastoid averaging, and Cz referencing. After the completion of pre-processing, data such as frequency and power were obtained, and the EEG signals in the α-band (8–13 Hz), β-band (13–18 Hz), and θ-band (4–7 Hz) were filtered out by wavelet transform. The mean energy of the EEG signal of the 25 test segments corresponding to the channels was extracted, and the energy of the data segment in the frequency band was represented by the logarithm of the sum of the squares of all data points in the frequency band with a base of 10, which is shown in Equation (1).

(1) $$E(k)=\mathrm{lg}\left[\sum_{i=1}^{n}x{\left(k\right)}_{i}{}^{2}\right],$$

where *k* represents the number of trials in the data segment (*k* = 1 in this experiment), *n* represents the number of data points in each segment, and $x{\left(k\right)}_{i}$ represents the value of the *i*th point in the *k*th data segment [31].

For the VEP, the same feature channels as the EEG statistical analysis were selected for statistical analysis (occipital region: POz, PO3, PO4, Oz, PO7, PO8). The data were filtered offline with a band-pass filter of 0.1–30 Hz, using the scene before the edit point as the baseline for each trial and selecting the first second of content after the start of the clip for analysis. VEP was calculated by averaging over trials and participants. Based on previous literature detecting time windows of interest [28], four time windows were selected to analyze ERP maxima on the scalp surface: Time window 1 = 140–190 ms after stimulus onset, Time window 2 = 180–220 ms after stimulus onset, Time window 3 = 250–380 ms after stimulus onset, and Time window 4 = 400–650 ms after stimulus onset.

### 2.5. Verification of Differences in VR Editing Methods Based on SVM

In order to further explore the most suitable frequency band for the classification of viewing load under the neural mechanism of the human brain, this paper adopts a support vector machine learning method to establish an SVM classification model based on EEG energy feature parameters to train and identify the energy induced by films with different VR editing methods for classification. Currently, the mainstream EEG classification methods include linear classifiers, such as support vector machines [36] and neural networks [37], among which SVM is the most widely used and effective classifier [9]. Although SVMs are binary classifiers, they can be used in multi-class problems by using a one-vs-one or one-vs-all strategy. Unlike neural networks, SVMs would not require a large number of training samples to solve the classification problem well. For linearly indistinguishable data, SVM can map to a high-dimensional feature space and find the optimal hyperplane in this space.

## 3. Results

In this paper, one-way repeated measures analysis of variance (ANOVA) was used for subjective and objective data, and simple effects analysis was performed if interactions between factors were found. All analyses were performed with *p* < 0.05 as the significance level measure, and the Greenhouse-Geisser method was used to correct degrees of freedom and *p* values. All statistical analyses were performed using SPSS 22.0 (IBM, Armonk, NY, USA).

### 3.1. Subjective Data

The NASA-TLX table evaluates the experimenter’s perceived load in six dimensions: Mental Demand (MD), Physical Demand (PD), Temporal Demand (TD), Effort (E), Performance (P), and Frustration Level (FL). The IPQ table consists of a three-factor structure of spatial presence (SP), involvement (INV), and reality (REAL). The level of load and immersion is expressed as the level of the scale score. For the subjective data, the questionnaire results of all volunteers are averaged and analyzed, and the statistical results are shown in Table 1.

**Figure 3:**
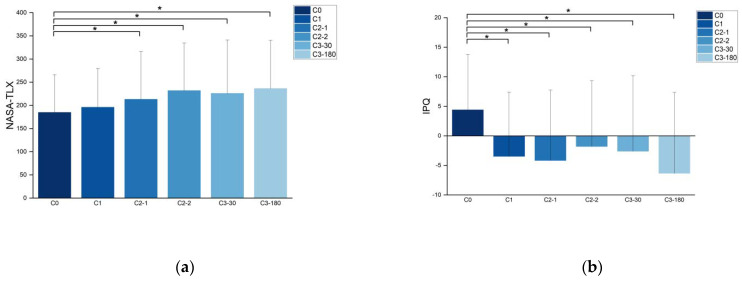
Subjective data. (**a**) NASA Task Load Index (NASA-TLX) total load mean; (**b**) immersion (IPQ) mean for each group, * indicates *p* < 0.05.

The analysis revealed significant image modality type grouping effects for both load (F(3.283,223.222) = 13.086, *p* < 0.001, χ^2^ = 98.018) and immersion (F(3.146, 91.247) = 7.822, *p* < 0.001, χ^2^ = 32.581) questionnaire results. For load, C0 stimuli evoked a significantly lower load than C2-1, C2-2, C3-30, and C3-180 stimuli evoked sensation (C1: *p* < 0.001, C2-2: *p* = 0.001 < 0.05, C3-30: *p* = 0.009 < 0.05, C3-180: *p* < 0.001). For immersion, C1 stimuli evoked significantly higher immersion than C1, C2-1, C2-2, C3-30, and C3-180 stimuli (C1: *p* < 0.001, C2-1: *p* = 0.002 < 0.05, C2-2: *p* = 0.012 < 0.05, C3-30: *p* = 0.002 < 0.05, C3-180: *p* < 0.001). In contrast, no effects of load and immersion were found between other groups (as shown in Figure 3).

### 3.2. Classification Results

The SVM-based VR editing method disparity validation experiment randomly divided the training and test sets in a 7:3 ratio for the number of samples [38]. The difference recognition effect of θ, α, β, θ + α, α + β, and θ + β band features on VR image presentation was compared, and the results are shown in Table 2. It can be observed that θ + β band features play the greatest effect on disparity recognition, with the classification accuracy of 92.590%, which is much higher than other groups. For the energy induced by movies with different VR editing methods, the recognition accuracy of β-wave band is much higher than other bands, reaching 87.012%, which is 34.821% higher than that of θ-wave band and 67.809% higher than that of α-wave band. This is similar to the results of Li et al. [39], who recorded EEG signals from 18 subjects to analyze the neurophysiological processes occurring during the code comprehension task and the possibility of distinguishing between expert programmers and beginners, and the results indicated that high-frequency bands such as β were the main feature. With this result, we further analyzed the EEG signals of each brain region corresponding to the theta and beta wave bands evoked by different conditions.

### 3.3. EEG Data Analysis

#### 3.3.1. Characterization of EEG Spectra in the Beta Wave Frequency Band

**Figure 4:**
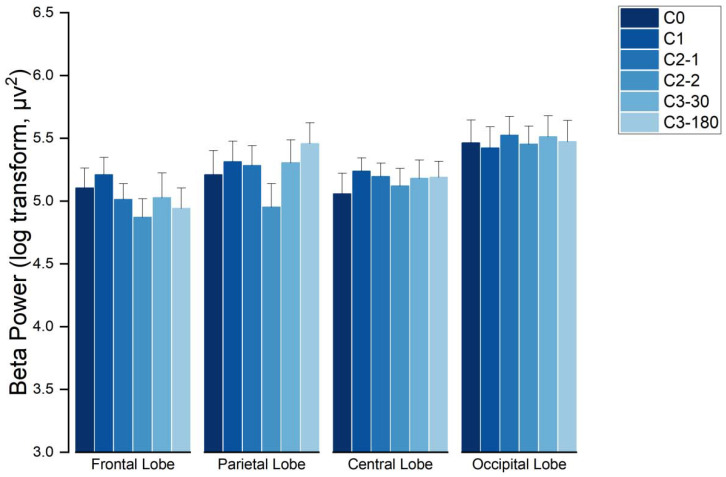
The distribution of EEG waves in brain regions with the beta wave frequency band.

For the beta wave frequency band, the interaction effect between brain functional regions—image pattern type group was investigated. Then, an analysis of variance for each brain functional region datum was performed with respect to the image pattern type group factor. The results showed that no effect of image pattern type grouping was found in the frontal, parietal, occipital, or central regions (as shown in Figure 4).

#### 3.3.2. Characterization of EEG Spectra in the Theta Wave Frequency Band

For the theta wave frequency band, the interaction effect between brain functional regions * image pattern type group was investigated. Then, an analysis of variance for each brain functional region data was performed with respect to the image pattern type group factor.

**Figure 5:**
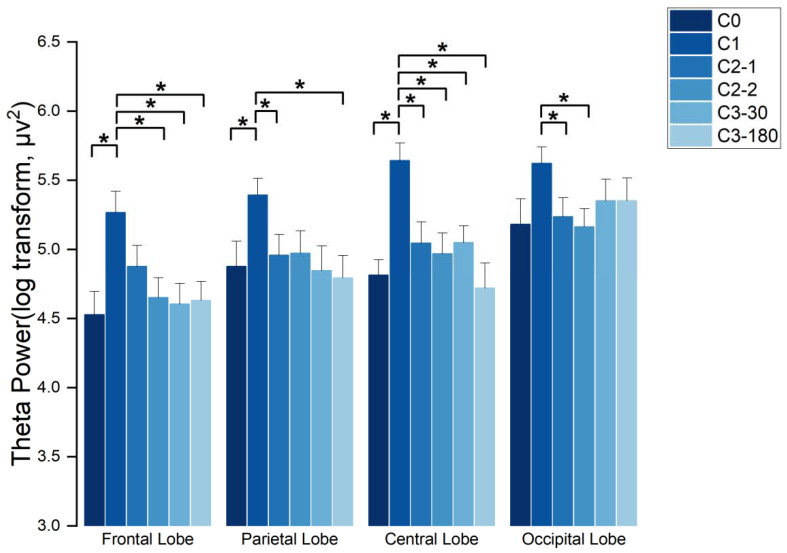
The distribution of EEG waves in brain regions with the theta wave frequency band, * indicates *p* < 0.05.

The results showed that there were significant effects in the frontal region (F(5145) = 6.738, *p* < 0.001, χ2 = 20.519), in the parietal region (F(5145) = 2.909, *p* = 0.16 < 0.05, χ2 = 28.324), in the central region (F(5145) = 8.650, *p* < 0.001, χ2 = 15.367), and in the occipital region (F(5145) = 2.538, *p* = 0.31 < 0.05, χ2 = 26.582), all with significant imaging pattern type grouping (Group) effects. The analysis showed that in the frontal region, the energy evoked by C1 stimuli is significantly higher than the energy evoked by C0 (*p* = 0.003 < 0.05), C2-2 (*p* = 0.002 < 0.05), C3-30 (*p* = 0.006 < 0.05), and C3-180 (*p* = 0.004 < 0.05) stimulus. In the parietal region, the energy evoked by C1 stimuli is significantly higher than the energy evoked by C0 (*p* = 0.025 < 0.05), C2-1 (*p* = 0.007 < 0.05), and C3-180 (*p* = 0.01 < 0.05) stimulus. In the central zone, the energy evoked by C1 stimuli is significantly higher than the energy evoked by C0 (*p* < 0.001), C2-1 (*p* = 0.006 < 0.05), C2-2 (*p* = 0.0005 < 0.05), C3-30 (*p* = 0.008 < 0.05), and C3-180 (*p* = 0.001 < 0.05) stimulus. In the occipital region, the energy evoked by C1 stimuli is significantly higher than those evoked by C2-1 (*p* = 0.035 < 0.05) and C2-2 (*p* = 0.0014 < 0.05) stimuli (as shown in Figure 5).

#### 3.3.3. Brain Topography

**Figure 6:**
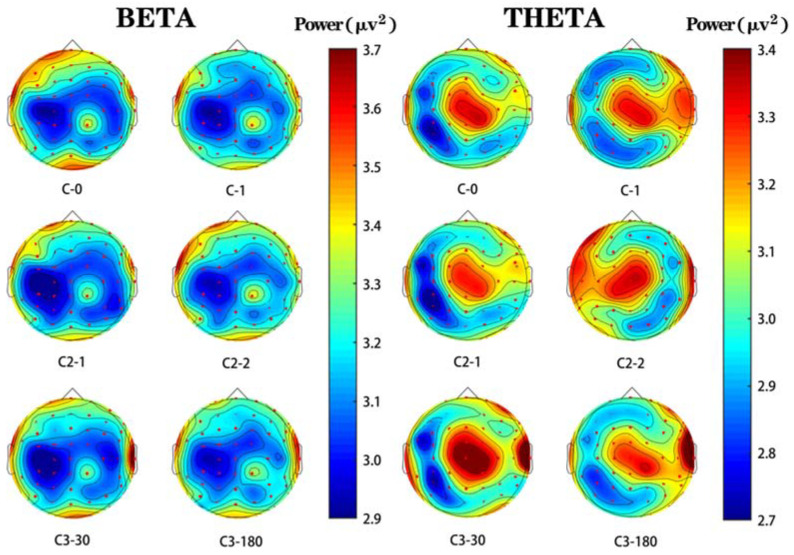
Brain topography in the beta- and theta-wave bands evoked by different images. Energy value is the average energy of all participants.

Figure 6 is a brain topographic map based on the average energy of all subjects. Among them, red indicates higher brain wave activity while blue indicates lower brain wave activity. According to the energy distribution of the brain topographic map, the energy induced by C0 stimulation is relatively low in the frontal lobe, parietal lobe, and occipital lobe, while the energy induced by C1 stimulation is relatively high in the same region.

Figure 6 is a brain topographic map based on the average energy of all subjects. Among them, red indicates higher brain wave activity, while blue indicates lower brain wave activity. According to the energy distribution of the brain topographic map, the energy induced by C0 stimulation is relatively low in the frontal lobe, parietal lobe, and occipital lobe, while the energy induced by C1 stimulation is relatively high in the same region.

#### 3.3.4. VEP Data Analysis

**Figure 7:**
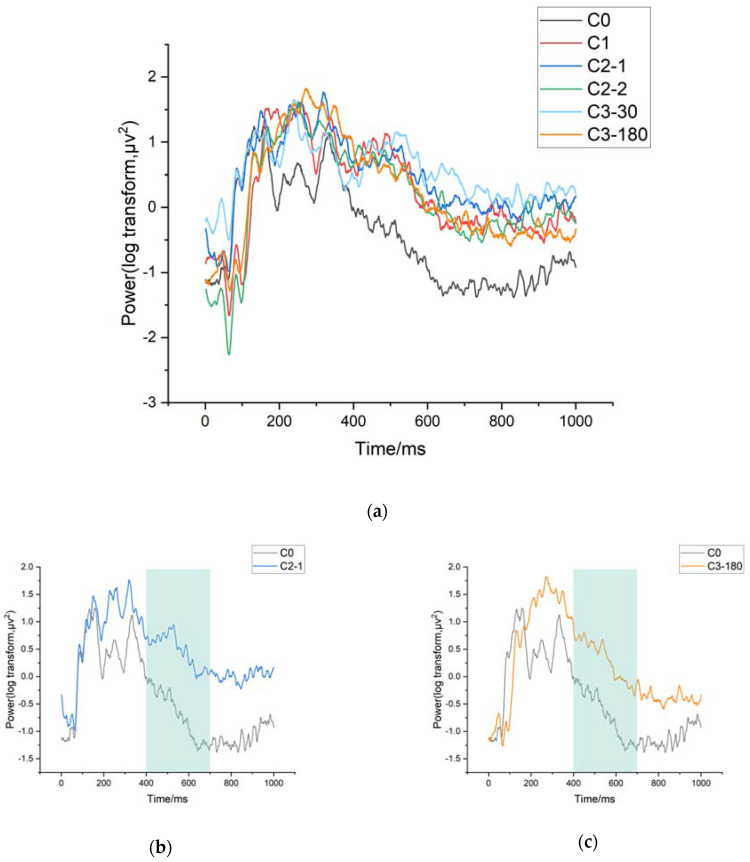
VEP Result. (**a**) VEP values for all clip methods; (**b**) C0 and C2-1 in regions with significant results; (**c**) C0 and C3-180 in regions with significant.

For each of the four selected time windows, an ANOVA analysis was conducted on the image pattern type grouping factor for each time window datum, in which the interaction effect time window-image pattern type group was investigated. The results demonstrated a significant image pattern type grouping effect in the fourth time window (F(5145) = 12.262, *p* < 0.001, χ2 = 11.537). No effect of image pattern type grouping was found in any of the remaining windows (as shown in Figure 7).

For window 4 (400–600 ms), energy values were significantly lower for C0 than for C-2-1 (*p* < 0.001) and C3-180 (*p* < 0.001). This indicates a significant difference between baseline and different clips, mainly in posterior regions, showing a late positivity.

#### 3.3.5. VEP Sources Analysis

The sLORETA traceability method in the LORETA software was used to compare the current density distribution and density intensity of brain activation areas under stimulation in two control groups (C0 and C2-1, C0 and C3-180), and the traceability results of P4-6 were analyzed for comparison.

**Figure 8:**
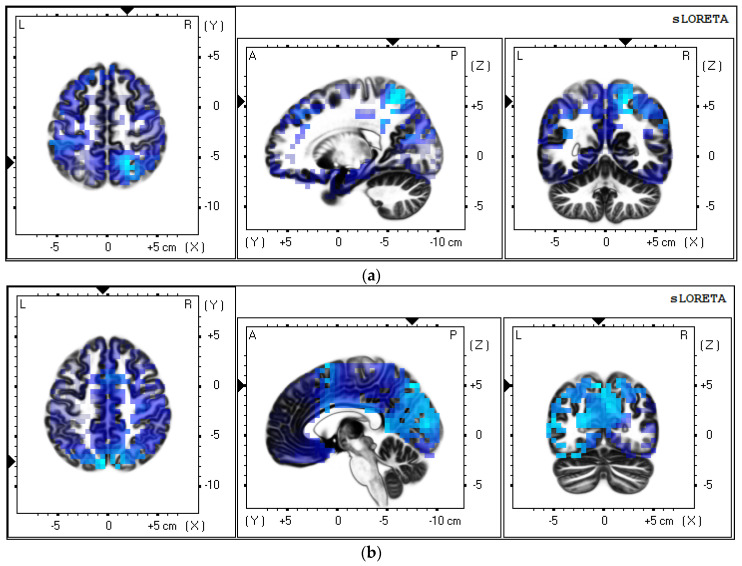
Schematic of current source density distribution. (**a**) C2-1; (**b**) C3-180.

As can be seen from Table 3 and Table 4 and Figure 8, the difference in current density of VEP sources compared to C0 and C1 is mainly in the precuneus. The difference in the current density of VEP sources compared to C0 and C2-1 is mainly in the inferior frontal gyrus. The difference in current density of VEP sources compared to C0 and C3-180 is mainly in the precuneus. The results indicate that the clip stimuli mainly responded to the higher cognitive areas, which could be the further processing of cognitive and visual information at the cognitive level, thus responding to the visual cortical areas of the human brain.

## 4. Discussion

### 4.1. Subjective Rating

For the trans-axial behavior of traditional images, George et al. demonstrated that this editing technique would confuse and disorient the viewer [5] but would not change the viewer’s enjoyment of the images. The NASA-TLX results show that C0 provides the least load perception and is statistically different from C2-1, C2-2, C3-30, and C3-108. Similar results could also be seen in the IPQ table. The results of the IPQ scale showed that C0 obtained the highest immersion score, and the differences among C1, C2-1, C2-2, C3-30, and C3-108 were all statistically significant. The C0 group had the highest immersion score and the lowest load perception score on the subjective scale. The other five editing methods reduced the immersion of VR images for viewers to a certain extent and were accompanied by a higher load perception. While C0 has the least effect on immersion and load perception, C-180 has the greatest effect, which is similar to the traditional editing method, i.e., trans-axis can also cause discomfort to viewers in VR images.

### 4.2. EEG Results

Frontal areas are associated with cognitive and motor functions, and parietal areas are associated with higher sensory processing, language functions, and spatial sense. The primary function of the temporal lobe is to process auditory stimuli, and the occipital area is the visual cortex, the main center for processing visual stimuli, and is also responsible for language, abstract concepts, and motor sensation [40]. A study of Ray William [41] showed that beta waves reflect emotional and cognitive processing in the brain. Increasing energy in the theta band of frontal areas is a marker for anxiety and situations requiring cognitive control [42]. Increasing beta-band energy responds to higher arousal and is associated with the increasing emotional intensity of alertness [43], attention [44,45], stress, anxiety, and agitation [46]. Changes in beta activity in the sensorimotor cortex are associated with sensorimotor control and peripheral muscle activity [47]. Experiments of Kosti et al. [48], on the other hand, demonstrated that theta and beta band energies were associated with cognitive effort, with a significant increase in arousal in beta and theta band energies in more complex tasks.

Compared to several other types of clips, unrelated clips of the film stimulated an increase in theta activity, especially in the frontal, parietal, and central regions. In addition, the occipital area, that is the visual cortex, was replaced by both the area with the largest differences and the frontal and central areas, which might be associated with higher cognitive and emotional processing, leading to greater differences. For the stimulation of images before and after the clips, the temporal, character, and spatial changes were manifested in a deeper processing of information at the cognitive level and visual information processing, thus responding to induce a greater degree of energy arousal in the visual cortex area, motor cortex area, and higher sensory processing area of the human brain. That is, for clips, similar movies are likely to be used, but the brain processes the content without “consciousness”. Furthermore, there were no significant differences within the C2 and C3 groups.

### 4.3. VEP Results

A study by Maffongelli exploring content and structure violations in action observation observed a late P4-6 in anterior regions following syntactic violations, associated with post-perceptual processes possibly serving an adjustment to the detected violation [49]. The results of this paper show that significantly higher potentials (P4-6) were produced in C2-1 and C3-180. These perceptions may be related to the belief that these changes may be of relevance to post-perceptual processes. Late P4-6 may be related to viewers’ thinking about their own perceptual conditions. For these common traditional editing methods, participants can be well adapted. However, for larger changes, such as a change of central characters in the film, audiences will consciously evaluate and make decisions about changes in video content.

### 4.4. VEP Sources Results

It has been shown that the increased mental load of working memory tasks is mainly manifested in multiple frontal and parietal lobes [50]. The results of the VEP source showed that the differences between the clips were mainly found in the frontal and parietal regions, rather than the occipital region, which is the visual processing area. It could be speculated that the change of character and the violation of the 180° rule of editing methods could bring a greater load to the audience. Meanwhile, the clip stimuli mainly responded to the higher cognitive areas, which might be the further processing of cognitive and visual information at the cognitive level, thus responding to the visual cortical areas of the human brain.

In summary, both subjective and objective data could confirm the disruption of the continuity of viewing by editing, but the impact of different editing methods varies, as the frontal and occipital lobes are more sensitive to changes in characters and changes in perspective. Viewers can accept temporal changes more naturally than spatial changes and are less likely to feel a sense of jumpiness and stress. Compared to the relevant clips, a film violating the 180° rule will cause a higher load on the viewer and a much less immersive viewing experience.

## 5. Conclusions

VR films form a different immersion and load from traditional films due to their unique presentation. While Neurocinematography is booming, using cognitive neuroscience to study the VR field has become a new trend. Through analyzing EEG energy features and subjective data on immersion and load, this paper focused on VR movie editing based on Neurocinematography, and used EEG energy features, elicit visual evoked potentials (VEP), and SVM’s viewing load classification model to compare different frequency bands of EEG for different editing recognition verification, and to investigate how traditional movie editing methods perform during the application in virtual reality and traceability of VEP data using the sLORETA traceability method. The results of subjective scales and objective data are similar. Since VR movies present 360° panoramic views, all editing methods would affect the perception of the virtual reality environment, producing stronger energy arousal. In the three dimensions of time, motion, and space, the change in motion had the least impact on the viewer, while the change in space had the greatest impact. Moreover, even if the presentation modes were completely different, the cognitive event segmentation theory was equally instructive for virtual reality editing, and viewers could understand VR films more naturally with relevant editing compared to irrelevant editing. In the comparison of cuts across the line events and long shot films, there was a significant difference in energy arousal, which was reflected in both the subjective and objective scales. However, the differences between clips are not felt in people’s consciousness but in higher cognitive areas, such as the prefrontal and parietal regions.

In order to avoid confounding factors and effectively control variables, the materials in this experiment were short videos. Therefore, some of the findings in this paper may not be applied outside of our study. Currently, studies on VR movie editing are relatively few. Thus, there are still many areas deserving in-depth study, such as the impact of elements, including emotion, duration, and storyline, on the viewer. Since the user’s viewing experience could be influenced by a variety of factors, a comprehensive study taking a wider variety of factors into consideration is necessary.

## Figures and Tables

**Table 1:** Participants’ load sense score statistics.

Group	Load		Immersion	
C0	184.72	81.385	4.400	9.3941
C1	195.86	83.635	−3.467	10.8841
C2-1	212.99	103.279	−4.167	11.9340
C2-2	231.94	102.753	−1.800	11.1244
C3-30	225.90	115.002	−2.600	12.7863
C3-180	236.20	104.177	−6.333	13.6895

**Table 2:** Accuracy results of classification under different frequency bands.

Classifier	Frequency Band	Accuracy
SVM	θ	52.191%
	α	19.203%
	β	87.012%
	θ + α	19.402%
	α + β	92.590%
	θ + β	22.948%

**Table 3:** Statistical comparison of VEP source current density between C0 and C2-1.

Talairach Coordinate(TAL)			Brodmann Area	Lobe	Structure
X	Y	Z			
−54	20	4	45	Frontal Lobe	Inferior Frontal Gyrus
−54	34	−2	47	Frontal Lobe	Inferior Frontal Gyrus

**Table 4:** Statistical comparison of VEP source current density between C0 and C3-180.

Talairach Coordinate(TAL)			Brodmann Area	Lobe	Structure
X	Y	Z			
−5	−70	50	7	Parietal Lobe	Precuneus

## Data Availability

The data presented in this study are available on request from the corresponding author. The data are not publicly available because we are creating an EEG data set.
